# *NF-YB*-Mediated Active Responses of Plant Growth under Salt and Temperature Stress in *Eucalyptus grandis*

**DOI:** 10.3390/plants10061107

**Published:** 2021-05-31

**Authors:** Jia-Hao Dai, An-Qi Hu, Jia-Shuo Zhang, Wen-Hai Liao, Hua-Yan Ma, Jin-Zhang Wu, Yuan Yu, Shi-Jiang Cao

**Affiliations:** 1 College of Forestry, Fujian Agriculture and Forestry University, Fuzhou 350002, China; daijiahao123@126.com (J.-H.D.); haqiii@163.com (A.-Q.H.); lwh1623850793@163.com (W.-H.L.); mhuayan97@163.com (H.-Y.M.); wjz253721079@163.com (J.-Z.W.); 2 Key Laboratory of Plant Molecular Physiology, CAS Center for Excellence in Molecular Plant Sciences, Institute of Botany, Chinese Academy of Sciences, Beijing 100093, China; zhangjiashuo20@mails.ucas.ac.cn; 3 University of Chinese Academy of Sciences, Beijing 100049, China; 4 FAFU-UCR Joint Center for Horticultural Biology and Metabolomics, Haixia Institute of Science and Technology, Fujian Agriculture and Forestry University, Fuzhou 350002, China; yyu@fafu.edu.cn; 5 College of Horticulture, Fujian Agriculture and Forestry University, Fuzhou 350002, China

**Keywords:** *NF-YB* gene family, *Eucalyptus grandis*, expression profiles, transcription factor, stress responses

## Abstract

The transcription factor NF-YB (nuclear factor-YB) family is a subfamily of the nuclear factor Y (NF-Y), which plays an important role in regulating plant growth, development and participates in various stress responses. Although the NF-Y family has been studied in many species, it is still obscure in *Eucalyptus grandis*. In this study, 23 *EgNF-YB* genes in eucalyptus were identified and unevenly distributed on 11 chromosomes. Phylogenetic analysis showed the *EgNF-YB* genes were divided into two clades, LEC-1 type and non-LEC1 type. The evolution of distinct clades was relatively conservative, the gene structures were analogous, and the differences of genetic structures among clades were small. The expression profiles showed that the distinct *EgNF-YB* genes were highly expressed in diverse tissues, and *EgNF-YB4/6/13/19/23* functioned in response to salinity, heat and cold stresses. Our study characterized the phylogenetic relationship, gene structures and expression patterns of *EgNF-YB* gene family and investigated their potential roles in abiotic stress responses, which provides solid foundations for further functional analysis of *NF-YB* genes in eucalyptus.

## 1. Introduction

NF-Y transcription factors (TFs), also known as the heme activating protein (HAP) and CCAAT binding factor (CBF), are common in advanced eukaryotes, such as yeast, animal and plant [1]. NF-Y usually consists of three different subunits, namely NF-YA (CBF-B or HAP2), NF-YB (CBF-A or HAP3) and NF-YC (CBF-C or HAP5) [2]. NF-YA, NF-YB and NF-YC form heterotrimeric complexes and interact with other regulatory factors to activate or inhibit the expression of downstream gene [3], and NF-YB plays an important role in the specific binding of the trimer to DNA [4]. The key domain of NF-YB subunit is homology to the conserved domain of histone folding motif (HFM) in core histone H2B [5,6] and plays a major role in protein-nucleic acid and protein-protein interactions [7]. NF-YB can be divided into three regions, A at the nitrogen end, B in the middle, and C at the carbon end, where A and C are non-conservative and B is a conservative domain family [8]. According to the homology comparison of B domains, *NF-YB* genes can be divided into LEC-1 type and non-LEC1 type. LEC-1 type includes LEC1 type and LEC1-LIKE, both of which have high homology [9]. The Asp-55 residue in the B domain of the LEC-1 NF-YB protein is the functional site and is associated with the regulation of seed embryo formation [10].

The numbers of NF-YB TFs found in different species are variable. In yeast and animals, NF-YB subunits are encoded by one or two genes [2,11]. Previous studies showed that various types of genes including transcription factors are replicated by the entire genome [12]. However, NF-YB subunits are expended and appear in the form of gene family in plants [13]. *AtNF-YB6* might have evolved from the genome of *Rosidae* and *MtNF-YB5* might have been derived from soybean [14]. This expansion is a common feature of the plant community, which makes *NF-YBs* specifically bind to the *cis*-acting factor of the target gene, activates or inhibits the expression levels of the downstream genes, thereby affecting the biological function in plants [15].

Different *NF-YBs* have distinct biological functions. A large number of studies have shown that *NF-YB* genes play an important role in plant development and widely participate in physiological and biochemical processes [16,17]. *NF-YBs* function in plant embryonic development, chloroplast formation and are involved in the regulation of photosynthesis, plant morphogenesis and flowering [1]. *AtNF-YB9* and *AtNF-YB6* participated in seed development by inducing the expression level of the genes that were related to embryo formation [18]. Meanwhile, the overexpression of *HvNF-YB1* induced early flowering [19]. *OsNF-YB7* played a role in the vegetative growth phase and regulation of floral meristem, thus delayed flowering and adjusted the plant height and number of grains [20]. The overexpression of *NF-YB* genes enhanced salinity, alkaline and drought tolerance, and antioxidant activity [21]. The transcriptome analysis of multi-species, including rice [17], maize [22], wheat [23], tung tree [24] and poplar [25] under dehydration treatments, further confirmed that the NF-YB members were involved in abiotic stress responses. Xu et al. [22] suggested that plant growth, development and stress adaptability are closely related to the differentiation of *NF-YB* genes function, which is a large number of elements related to abiotic stress in nature promoter.

*Eucalyptus grandis*, a species of eucalyptus in *Myrtaceae*, is a significant fast-growing broad-leaved tree species [26]. Eucalyptus together with poplar and *Pinus* are considered to be the three fast-growing tree species in the world [26]. *E. grandis* has the advantages of rapid growth, high yield and quality, strong resistance to adversity, strong germination ability and high economic value, which can bring huge economic, ecological and social benefits, and it also has a very obvious development. However, abiotic stresses such as low temperature and salt stress often affect the growth of *E. grandis*, resulting in a decrease in yield [27,28]. Therefore, it is of great significance to study and analyze the genes related to stress response for the improvement of fine traits and yield in *E. grandis*.

Although many researchers have studied the *NF-YB* gene family in various plant species, the eucalyptus *NF-YB* gene family has not been systematically explored. In this study, bioinformatics methods were used to identify and analyze the physical and chemical properties of protein sequence, the construction of phylogenetic tree, gene chromosome location, gene structure analysis and conservative domain of the 23 *NF-YB* members in eucalyptus. Besides, phylogenetic relationships, gene structure and motif composition of *NF-YB* genes and the expression pattern of *NF-YB* genes under different eucalyptus tissues and various abiotic stress conditions were also studied.

## 2. Results

### 2.1. Phylogenetic and Evolutionary Analysis of EgNF-YB 

**Figure 1:**
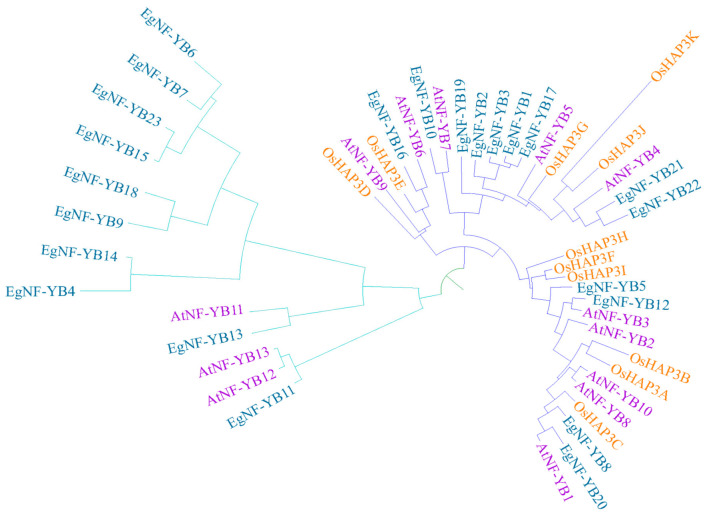
Phylogenetic classification of *NF-YB* genes in *Eucalyptus grandis*, *Arabidopsis* and *Oryza sativa*.

The evolutionary relationship of a gene family plays a significant role in predicting and verifying the function of the gene family. We downloaded the *AtNF-YB* and *OsNF-YB* (also known as HAP3 [29]) protein sequences from phytozome 12. Then we downloaded the HMM profiles of NF-YB domain (PF00808) as a reference to predict *EgNF-YB* protein in the eucalyptus genome sequence via HMMER software 3.0 with a threshold of e-value < e^−5^. A total of 23 candidate genes were identified in eucalyptus, named from *EgNF-YB1* to *EgNF-YB23*, and further used for subsequent analysis (Table S1). We constructed an un-root phylogenetic tree with 23, 13 and 11 *NF-YB* genes from eucalyptus, *Arabidopsis* and rice, respectively, in order to understand the phylogenetic relationship and evolutionary history of *EgNF-YBs*. In the phylogenetic tree, *EgNF-YB* genes were clustered into two distinct subgroups, named LEC-1 (13 genes) and non-LEC1 (10 genes) (Figure 1 and Figure S1). The *EgNF-YB* genes in the same subgroup may have similar functions, such as abiotic stress response or flowering time [29]. Multiple alignments of *EgNF-YB* family members showed that *EgNF-YBs* were highly conserved. There was one completely conserved site (tryptophan) among the 23 *EgNF-YB* genes. Besides, four α-helix regions were found in *EgNF-YBs*. Although individual amino acids were different, the core functional amino acids were highly conserved (Figure S2).

### 2.2. Classification and Structural Analysis of EgNF-YB

**Figure 2:**
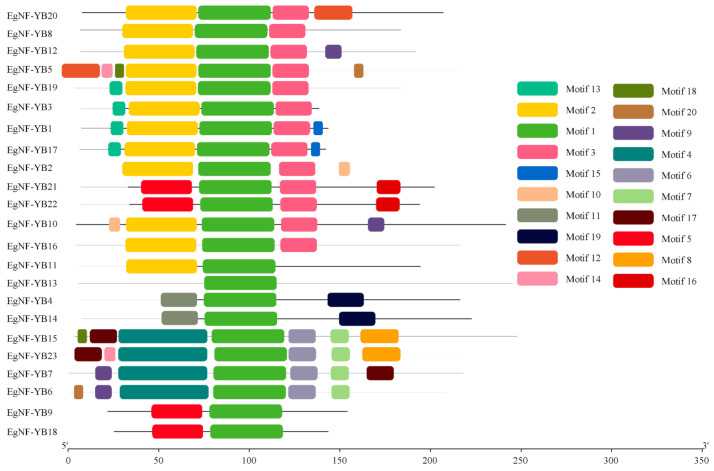
The conserved motifs of *EgNF-YB* genes. The conserved motifs in the EgNF-YB proteins were identified with MEME software. Black lines denote the non-conserved sequences, and each motif is indicated by a colored box numbered on the right. The length of motifs in each protein is presented proportionally.

The Multiple Expectation-Maximization for Motif Elicitation (MEME) software and Gene Structure Display Server (GSDS) online service were used to investigate the classification and gene structure of the *EgNF-YB* family in eucalyptus. We determined the conserved motif and gene structure of EgNF-YB proteins and constructed a phylogenetic tree. There were 20 motifs in the *EgNF-YB* family. Motif 1 was observed in all *EgNF-YB* genes, indicating that motif 1 plays a crucial role in eucalyptus. While motif 3, a histone-like TF (CBF/NF-Y), was specifically identified in the LEC-1 type. Other motifs appeared irregularly in other genes. Therefore, motif 1 and motif 3 were suggested as the core motifs of the *NF-YB* family. Interestingly, *EgNF-YB13* only contained motif 1, suggesting that it may be involved in unique features (Figure 2).

**Figure 3:**
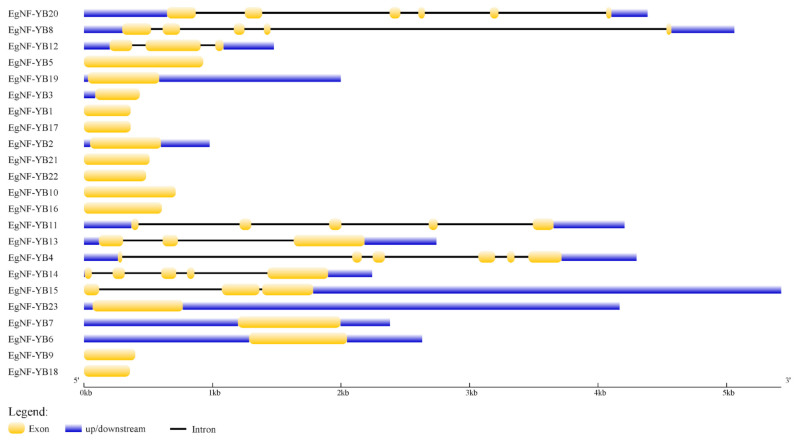
Exon-intron gene structures of *EgNF-YB*. Gene structure analysis was performed using GSDS. Length of exons and introns for each *EgNF-YB* gene is proportionally displayed.

In order to understand the structure of the 23 *EgNF-YB* genes, we analyzed them using GSDS online service [30] to find out the conserved domains. The results showed that the gene structure of the *EgNF-YBs* was multilateral. Nine genes (*EgNF-YB1*, *EgNF-YB5*, *EgNF-YB9*, *EgNF-YB10*, *EgNF-YB16*, *EgNF-YB17*, *EgNF-YB18*, *EgNF-YB21* and *EgNF-YB22*) contained only exons, and the other 14 genes included introns and untranslated regions (Figure 3). Introns were significantly degraded during evolution, which may be related to the function of genes. Fewer introns can initiate transcription, thereby accelerating the expression or inhibition of related genes [31]. In terms of the number of introns, 15 genes did not contain introns, implying that they may be involved in some rapid response processes. Three genes (*EgNF-YB12*, *EgNF-YB13* and *EgNF-YB15*) contained two introns, three genes (*EgNF-YB8*, *EgNF-YB11* and *EgNF-YB14*) contained four introns, and two genes (*EgNF-YB20* and *EgNF-YB4*) contained more than five introns (Figure 3). It is suggested that the various gene structures of the *EgNF-YBs* family may lead to a variety of biological functions.

### 2.3. 3D Structure Analysis of EgNF-YBs

**Figure 4:**
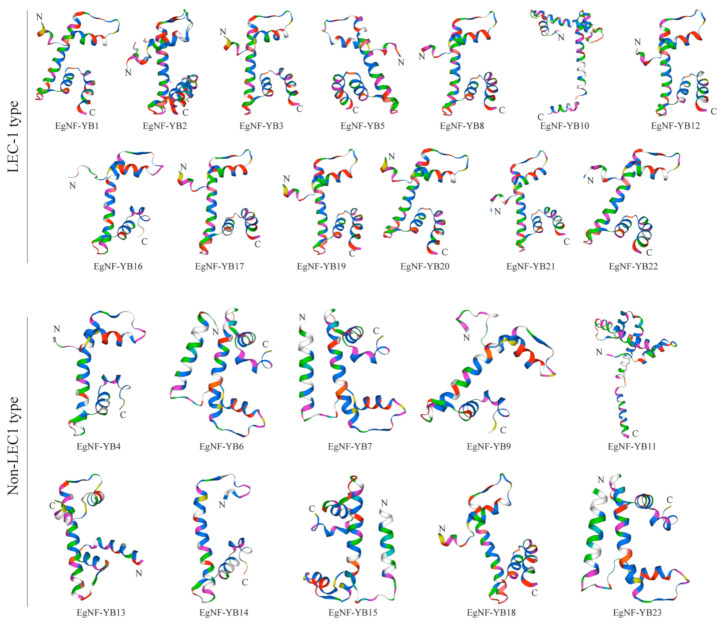
Homology modeling of NF-YB protein in *Eucalyptus grandis*. The 3D structure of NF-YB protein was predicted based on the Swiss-model database.

To better analyze the protein three-dimensional (3D) structure of *EgNF-YB* family, we used the online prediction website SWISS-MODEL [32,33] to draw up the 3D structure of EgNF-YB protein (Figure 4). In the whole family, the secondary structure mainly existed in the form of α-helix and random curl [34]. In general, the 3D structure of the LEC-1 type protein was significantly simpler than that of the non-LEC1 type. The structures of LEC-1 type members were highly similar. There was one α-helix and several random curls, indicating that the function of LEC-1 type members may be highly similar. The structure of non-LEC1 type differed from that of LEC-1 type and was more complex. Among non-LEC1 type genes, *EgNF-YB6/7/15/23* contained two helices, while others had only one helix. It is proposed that the functions of the two types of genes may be altered, and the roles of the non-LEC1 type genes are more diverse. Since the protein structure determines its function, we can propose that the non-LEC1 type genes of *EgNF-YB* family play a more significant role in plant growth and development in eucalyptus.

### 2.4. Chromosome Localization of EgNF-YB Genes

**Figure 5:**
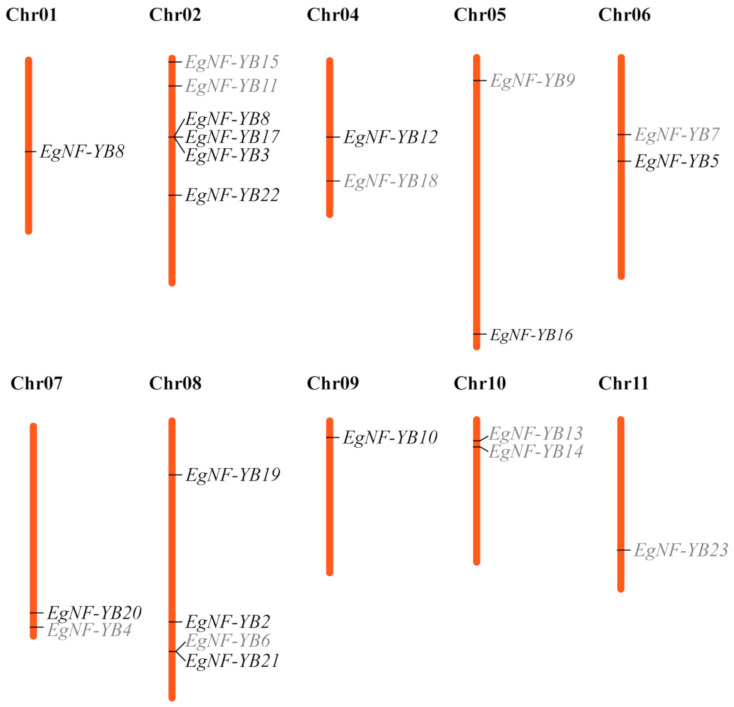
Chromosomal localization of *EgNF-YB* genes on the 11 eucalyptus chromosomes. The number of chromosomes is displayed at the top of each vertical bar. The corresponding *EgNF-YB* gene names are connected by a short line on the left side, and different colors represent different subfamilies. Black is the LEC-1 type, and grey is the non-LEC1 type.

*EgNF-YB* genes are widely distributed in the reference genome of eucalyptus. However, chromosome 3 did not contain any *EgNF-YBs*. Chromosomes 1, 9, and 11 all had one gene of *EgNF-YB* family, while the remaining seven chromosomes each contained two to six genes. It is worth mentioning that there were only LEC-1 type genes (*EgNF-YB13*, *EgNF-YB14* and *EgNF-YB20*) identified on chromosomes 10 and 11. A large number of *EgNF-YB* genes were observed on chromosome 2, including four non-LEC1 type genes (*EgNF-YB1*, *EgNF-YB3*, *EgNF-YB17* and *EgNF-YB22*) and two LEC-1 type genes (*EgNF-YB11* and *EgNF-YB15*). In addition, *EgNF-YB6* and *EgNF-YB21* were clustered on chromosome 8, indicating possible tandem duplications. Interestingly, chromosomes 4, 5, 6 and 7 each contained one LEC-1 type and one non-LEC1 type gene. Generally, *EgNF-YB* genes were more evenly distributed in eucalyptus (Figure 5).

### 2.5. Expression Pattern of Eucalyptus EgNF-YB Genes in Various Tissues

**Figure 6:**
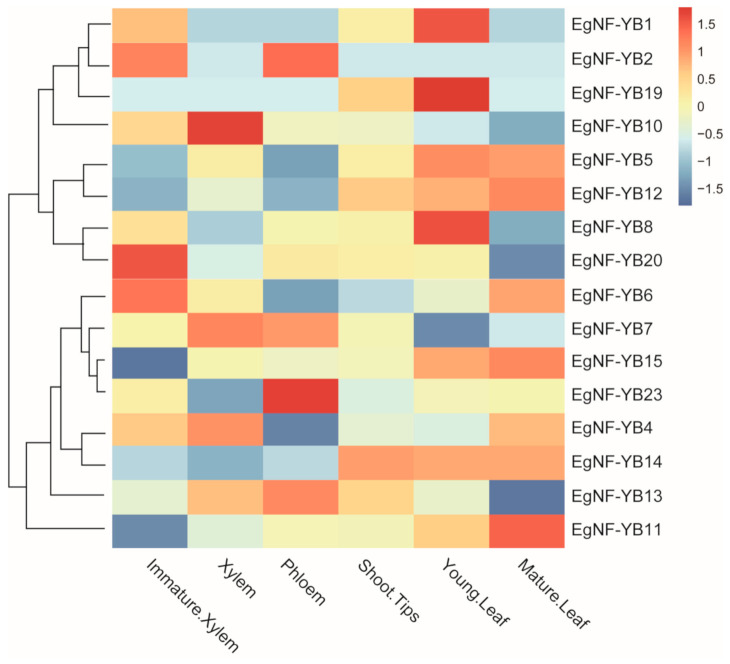
Expression patterns of 16 *EgNF-YB* genes in immature xylem, xylem, phloem, shoot tips, young leaf and mature leaf of eucalyptus. Different colors in the map represent gene transcript abundance values as shown in the bar by the side of the figure.

To further understand the function of *EgNF-YB* genes in eucalyptus, we investigated the expression levels of *EgNF-YBs* in different tissues including immature xylem, xylem, phloem, shoot tips, young leaf and mature leaf based on the fpkm values. As shown in Figure 6, the expressions of *EgNF-YB13* and *EgNF-YB20* in young leaves were moderate but decreased rapidly after leaf maturation, which may reveal that *EgNF-YB13/20* genes are involved in leaf development. The expression levels of *EgNF-YB2* and *EgNF-YB20* increased slowly from phloem to immature xylem and then decreased at xylem. This result suggested that *EgNF-YB2/20* may function in the process of xylem formation and differentiation. Interestingly, the expression levels of 23 genes in the shoot tips were similar, and most of the *EgNF-YB* genes were highly expressed in young leaves, especially *EgNF-YB19*, *EgNF-YB1* and *EgNF-YB8*. It can be suggested that the *EgNF-YB* genes play a crucial role in the physiological activities of young leaves.

### 2.6. EgNF-YB Genes Response to Salinity and Extreme Temperature Stress

**Figure 7:**
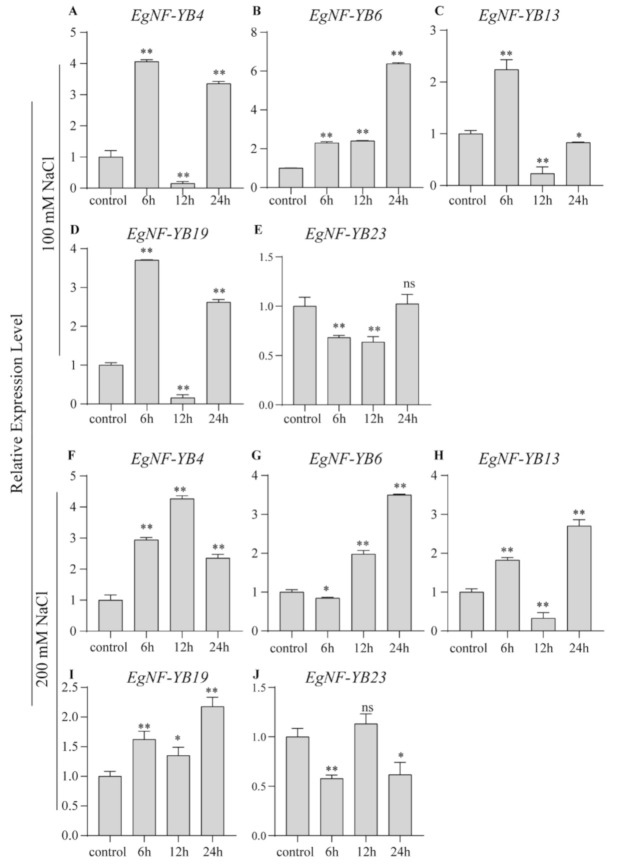
qRT-PCR expression analysis of five selected *EgNF-YBs* genes under salt treatment. (**A**–**E**) low salt (100 mM NaCl); (**F**–**J**) high salt (200 mM NaCl). The processing time of each group was control (no treatment), 6 h, 12 h and 24 h. Mean expression value was calculated from three independent replicates. Error bars indicate standard deviation. Data are presented as mean + standard deviation (SD). Asterisks on top of the bars indicate statistically significant differences between the stress and counterpart controls (* *p* < 0.05, ** *p* < 0.01 and *ns* > 0.05).

Previous studies have shown that NF-YB transcription factors are involved in regulating the tolerance of different abiotic stresses in many plant species such as *Arabidopsis* [35] and rice [36]. However, there were no transcriptomic data of eucalyptus related to salt stress. Salinity is one of the abiotic stress factors that seriously reduce the economic value of eucalyptus. In order to explore whether *EgNF-YBs* can respond to salt stress, we used q-PCR to explore the expression levels of five *EgNF-YBs* stimulation with low salt (100 mM NaCl) condition or high salt (200 mM NaCl) stimulation. Under the low salt treatment, the mRNA level of *EgNF-YB4* increased rapidly by 4.1-fold than that under the normal conditions, and then decreased sharply, and increased again at 24 h (Figure 7A). However, in high salt condition, the transcription level of *EgNF-YB4* increased continuously and reached a maximum at 24 h (Figure 7F). It is suggested that *EgNF-YB4* functions in a high salt environment. Interestingly, the expression level of *EgNF-YB13* and *EgNF-YB19* showed a similar trend (Figure 7C,D,H,I). 

**Figure 8:**
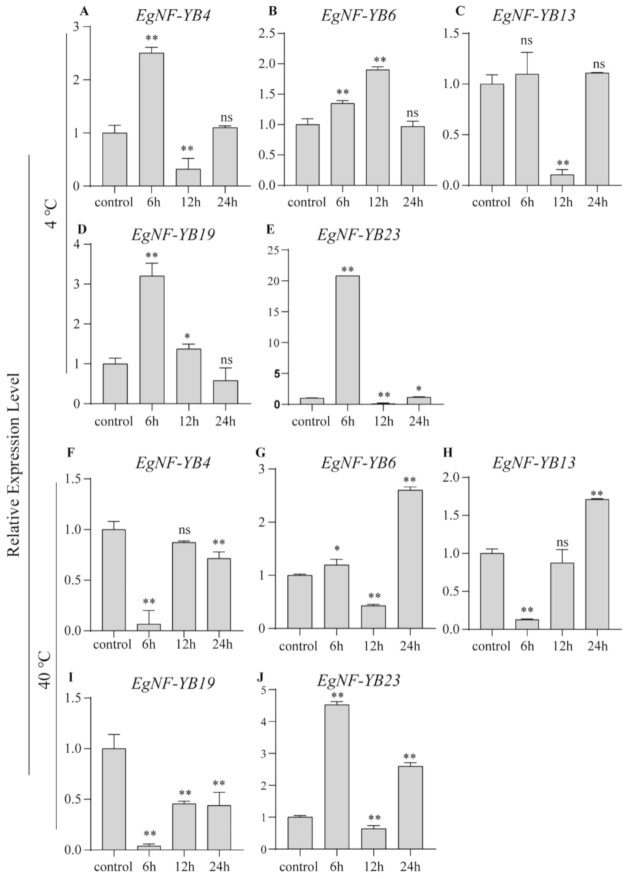
qRT-PCR expression analysis of five selected *EgNF-YBs* genes under temperature treatment. (**A**–**E**) low temperature (4 °C); (**F**–**J**) high temperature (40 °C). The processing time of each group was control(no treatment), 6 h, 12 h and 24 h. Mean expression value was calculated from three independent replicates. Error bars indicate the standard deviation. Data are presented as mean + standard deviation (SD). Asterisks on top of the bars indicate statistically significant differences between the stress and counterpart controls (* *p* < 0.05, ** *p* < 0.01 and *ns* > 0.05).

The transcription level of *EgNF-YB6* differentiated at 6 h under low and high concentrations of salinity stimulation, then gradually accumulated and reached the maximum at 24 h, which was 6.4-fold and 3.5-fold of that at 0 h, respectively (Figure 7B,G). The results showed that the function of *EgNF-YB6* was more conservative in response to salt stress and did not depend on salt concentration. Compared with the control group, the mRNA level of *EgNF-YB23* decreased slowly at low salt, and there was no significant difference with the control treatment at 24 h (Figure 7E). Under the high salt condition, the expressions of *EgNF-YB23* floated in waves (Figure 7J). In general, *EgNF-YB23* may play a negative role in the early stage of salt stress response. Overall, *EgNF-YB4*, *EgNF-YB6*, *EgNF-YB13*, *EgNF-YB19* and *EgNF-YB23* can respond to salt stress signals.

q-PCR results showed that the expression level of the five genes varied under high or low temperatures. Compared to the control group, the transcriptional levels of *EgNF-YB4*, *EgNF-YB19* and *EgNF-YB23* reached the highest at 6 h under low-temperature stress (Figure 8A,D,E). However, the mRNA level of *EgNF-YB23* accumulated gradually at 6 h and 12 h and decreased to the original transcriptional level at 24 h (Figure 8B). *EgNF-YB13* was only reduced at 12 h, and there was no significant difference in other detection time points compared with the control group (Figure 8B). Under high temperature, the transcriptional levels of *EgNF-YB6*, *EgNF-YB13* and *EgNF-YB23* got the highest at 24 h, 24 h and 6 h, respectively. The expression level of *EgNF-YB19* was always lower than that of the control group. Our results may provide some clues for further functional study of *EgNF-YBs* in abiotic stress responses.

## 3. Discussion

Plants are facing major challenges from biotic and/or abiotic stresses. Transcription factors respond to various stresses via regulating target gene expressions [37]. NF-YB transcription factor family exists widely in eukaryotes and can activate or suppress the transcription levels of target genes by binding to CCAAT-box [38,39], and play an important role in biotic and/or abiotic stress response [16]. Eucalyptus is an essential economic tree with fast growth and high economic values. So far, NF-YB transcription factors have been widely studied in several species but remain largely uncharacterized in the eucalyptus. 

In this study, 23 candidate *EgNF-YB* genes were identified in eucalyptus using bioinformatics, and the annotation was carried out via homologous genes of *Arabidopsis* and rice. We found that eucalyptus had 10 and 12 *NF-YB* members more than *Arabidopsis* and rice, respectively (Figure 1). The *EgNF-YBs* family in eucalyptus have different evolutionary branches. Moreover, our results also showed that the members of *EgNF-YBs* family in rice, *Arabidopsis* and eucalyptus were crisscross distributed and did not form any separate branches (Figure 1). It demonstrates that the *NF-YB* families of monocotyledons and dicotyledons are still conservative in evolutionary direction, and there was no obvious distinguish [40]. 

Based on topological structure, the *EgNF-YB* gene family in eucalyptus was divided into two subgroups, the LEC-1 type and the non-LEC1 type (Figure S1). The LEC-1 type included 13 *EgNF-YB* members, accounting for 56.52% of the total number, and the non-LEC1 type included 10 *EgNF-YB* members (Figure S1). The phylogenetic prediction of those genes showed that the *EgNF-YB* family members of eucalyptus might be expanded in some unknown functional groups [25,41]. It is suggested that *EgNF-YB* genes may be more important in perennial eucalyptus. However, due to the lack of characterization of *EgNF-YBs* gene function in eucalyptus, further analysis is needed to understand whether the *EgNF-YB* genes are associated with the perennial characteristics of eucalyptus, or whether they are just redundant or backup genes or other functions [42,43]. 

*EgNF-YB* motifs analysis showed that motif 1 was significantly conserved in almost all *EgNF-YB* genes, which can be used as a symbol of the NAC domain. In addition, motif 2 and motif 14 appeared in most LEC-1 type *EgNF-YB* genes, suggesting that it may be the key domain to distinguish LEC-1 type from non-LEC1 type (Figure 2). Interestingly, the number and type of motifs also represent the structural basis of gene functional diversity. Notably, *EgNF-YB15* and *EgNF-YB23* had the most diverse motifs (7 different motifs). They were clustered in the same branch and might have similar functions (Figure 2). The motif 1 of the eucalyptus *NF-YB* genes was highly similar to the DNA binding domain (Figure 2), suggesting that motif 1 may bind to CCAAT-box [17]. Few introns were observed in some *EgNF-YBs* (Figure 3), indicating that the faster velocity EgNF-YB TFs can bind to CCAAT-box to activate or inhibit the transcription level of target genes [31]. Based on the functional domain of NF-YB protein, we can speculate that CBF-NFYB-HMF domain of protein structure is a highly conserved region of LEC1 and L1L proteins [44], and the differentiation degree of CBF-NFYB-HMF domain is very low in evolution. Multiple sequence alignment of conserved regions of EgNF-YB family proteins showed that EgNF-YBs contained four α domains (Figure S2), which are necessary for EgNF-YBs to perform DNA binding and interact with NF-YC to form heterodimers and other proteins [35]. The number of introns can represent the original genes [45], implies that *EgNF-YB20*, *EgNF-YB4* and *EgNF-YB14* may represent the original genes of *EgNF-YBs* in eucalyptus (Figure 3). Intron numbers are closely related to the expression level of genes as well [31,46]. Similarly, the evolutionary relationship between similar intron-exon structure and length was clustered. This revealed that each branch had a conservative number of motifs and/or length of introns-exon, which supported the close evolutionary relationship between them and the classification of subgroups (Figure 2, Figure 3 and Figure S1).

*EgNF-YB* genes were unevenly distributed on 11 chromosomes of eucalyptus, which may be due to the complex gene rearrangements and deletions. Chromosome mapping analysis showed that non-LEC1 type genes were distributed on eight chromosomes of eucalyptus, indicating that the basic characteristics of this type of genes form earlier and are relatively independent, while LEC-1 type genes had joint phenomenon, which affected their expression (Figure 5). All of these are favorable for the family genes to play an important regulatory role in the stress response and for the expression of stress resistant genes.

**Figure 9:**
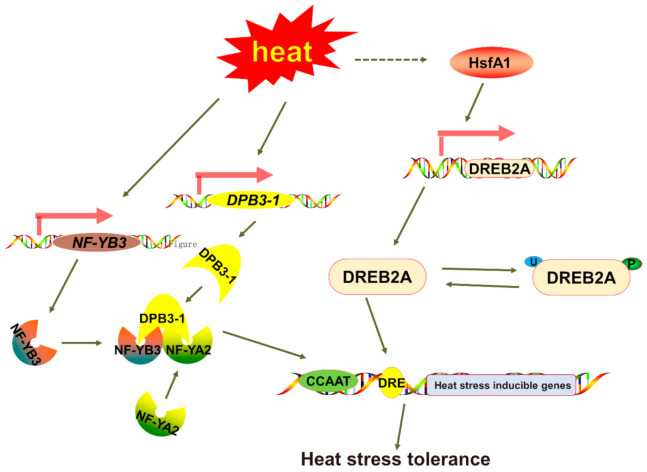
A model of the function of NF-YB3 under heat stress. The *NF-YB3*, *DREB2A* and *DPB3-1* genes are induced by heat stress. The NF-YB3, NF-YA2, and DPB3-1 form a trimer in the nucleus. Thereafter, the trimer enhances the efficiency of transcription by DREB2A. The NF-YB2 is suggested to activate the heat inducible genes through CCAAT elements and the DREB2A activates the target genes through DRE elements, which results in the heat stress tolerance.

Previous studies showed that the *NF-YB* genes play an significant role in plant development, including late embryogenesis, flowering time, drought tolerance, etc. [35]. In our study, we found that *EgNF-YB* family members presented diverse expression patterns in different tissues (Figure 6). In the immature xylem, *EgNF-YB20* expressed the highest mRNA level, but not in the mature leaves. Woody biomass is widely used in pulp, paper and chemical cellulose industries [47]. Whether *EgNF-YB10*, which was specifically abundant in xylem (Figure 6), can promote xylem development and improve the economic value of eucalyptus is also worthy of further study. *EgNF-YB2/23* were highly expressed in the phloem, suggesting that they may participate in assimilation transport in response to biotic or abiotic stress. Interestingly, under high temperature or low temperature, the transcription level of *EgNF-YB23* had dynamic changes (Figure 8E,J), which also implies that *EgNF-YB23* may be involved in the transport of assimilates between leaves and phloem. In general, we did not find a certain *EgNF-YB* gene that was stably expressed in multiple tissues, which indicates that *EgNF-YBs* are involved in the growth and development of specific tissues. The diverse expression patterns of *EgNF-YBs* show that they may have distinct functions. In our study, the expression patterns of *EgNF-YB* genes indicate that they may be associated with the response to abiotic stress, including high or low salt stress and hot or cold stress (Figure 7 and Figure 8). Taking 40 °C as an example, it’s speculated that *EgNF-YB6/23* may have some interaction or influence with some specific regulatory factors in the early response process. *EgNF-YB4/13/19*, however, play a slow role in the downstreams, which may result in two different expression patterns in response time point. Therefore, our results show that EgNF-YB TFs play an important role not only in many biological processes of eucalyptus development (such as xylem and phloem development), but also in the stress tolerance pathways of eucalyptus such as low/high temperature (Figure 9) [48,49,50] and salt stress. 

We analyzed the *EgNF-YB* gene family of eucalyptus and revealed that the *EgNF-YBs* participated in a variety of biological processes, which provides an important theoretical basis for further studying the function of the *EgNF-YBs* and speeding up the practical application of this family, and presents a new insight for excavating the function and further accelerating the application of *EgNF-YBs*.

## 4. Materials and Methods

### 4.1. Identification and Chromosomal Location of NF-YB Family Members in Eucalyptus

We downloaded NF-YB protein sequence of Arabidopsis and rice (*Oryza sativa*) from Phytozome (https://phytozome.jgi.doe.gov/pz/portal.html, accessed on 6 May 2021). Downloaded the Hidden Markov model (HMM) file corresponding to NF-YB domain (PF00808) from Pfam protein family database (http://pfam.sanger.ac.uk, accessed on 6 May 2021), a threshold of e-valus < e^−5^ [51]. we used SMART (Simple Molecular Agricultural Research Tool, http://smart.emblheidelberg.de, accessed on 6 May 2021) to verify the candidate genes, integrate all the obtained protein sequences and remove the wrong sequence by manual screening. A total of 23 EgNF-YB genes were identified in the eucalyptus genome and those candidate genes contained NF-YB domains for further analysis. Then the chromosome location of EgNF-YB genes was obtained from the genome annotation information of plant zombie v12.1.6 (https://phytozome.jgi.doe.gov/pz/portal.html, accessed on 6 May 2021) and utilized MapChart to visualize the location and length of the corresponding chromosomes.

### 4.2. Gene Duplication and Phylogenetic Analysis

The protein sequences of eucalyptus, *Arabidopsis* and rice *NF-YBs* were compared by MUSCLE v3.8.31 [52]. We used the Maximum likelihood module of IQ-Tree in the PhyloSuite software to generate phylogenetic trees [53]. The resulting chart was the EvolView online service (https://www.evolgenius.info/evolview, accessed on 6 May 2021).

### 4.3. Analysis of NF-YB Gene Structure and Protein Conserved Motif

We used the online service of the Gene Structure Display Server (GSDS) (http://gsds.cbi.pku.edu.cn, accessed on 6 May 2021) [30,54] to visualize the number and distribution of exons and introns. The GFF annotation file of *NF-YB* genes was downloaded from Phytonome, and the annotation of *EgNF-YB* genes was uploaded to GSDS website according to the default parameters of exon-intron structure of *EgNF-YB* genes. We utilized the Multiple Expectation-Maximization for Motif Elicitation (MEME) software [55,56] to find the conserved motif of EgNF-YB proteins in eucalyptus, and further determined the composition of neoconservative bases that may not be recorded in the public database. The parameters used were as follows: the maximum base order width was 100aa, the number of motifs was 10, and other options were the default values. We used TB tools [57] to modify it and confirm the type of motifs, we also deduced the three-dimensional (3D) structure of eucalyptus NF-YB protein in the SWISS-MODEL database (https://swissmodel.expasy.org/interactive, accessed on 6 May 2021).

### 4.4. Tissue Expression Profile Analysis of NF-YB Genes

We downloaded RNA-Seq data from Phytozome [58], including different tissues from different developmental stages: immature xylem, xylem, phloem, shoot tips, young leaf and mature leaf. We used the R pheatmap (https://CRAN.R-project.org/package1⁄4pheatmap, accessed on 6 May 2021) software package to draw the thermal map of genes to show the expression map of *EgNF-YB* genes in different tissues and developmental stages.

### 4.5. Plant Materials and Abiotic Treatment

Eucalyptus (*Eucalyptus grandis*, Eg5) one-year-old seedlings were collected from the forestry department of Fujian Agriculture and Forestry University (119°14′ E, 26°5′ N) and grown on local soil under natural conditions. The relative humidity was about 77% and the light intensity was 150 μmol·m^−2^·s^−1^. Seedlings were then exposed to salt stress (100 mM or 200 mM NaCl) or cold stress (4 °C) or heat stress (40 °C). Samples were collected at 0, 6, 12 and 24 h, respectively. The control group was in normal growth conditions. Samples of leaves were harvested after treatment and stored at −80 °C for RNA isolation.

### 4.6. RNA Extraction and Quantitative Real-Time PCR 

Total RNA from the samples under different stress treatment was extracted using the OMEGA plant RNA Kit (Omega Bio-Tek, Shanghai, China) and following the operating instruction. cDNA synthesis was followed by Takara’s PrimeScript Synthesis 1st Strand cDNA Synthesis Kit (Takara, Beijing, China). Quantitative real-time PCR analysis was performed with TransStart ^®^Top Green qPCR SuperMix (Transgen, Beijing, China) and a Bio-Rad CFX-96 detection system, as follows: 94 °C for 30 s, then 45 cycles of 94 °C for 5 s, 60 °C for 1 s. β-actin was used as a reference gene. All primers used for q-PCR were listed in Table S2. The raw Ct data were analyzed by 2^−∆∆Ct^ [59]. Three technical replicates were performed for each three independent biological replicates.

## 5. Conclusions

In summary, a total of 23 eucalyptus *EgNF-YB* genes were characterized, which were distributed on 11 different chromosomes. This study used bioinformatics methods to perform phylogenetic tree analysis, gene structure analysis, conserved motif identification, chromosomal localization, and expression pattern of the *EgNF-YB* genes in different tissues and q-PCR results showed the different abiotic stress responses. This study lays a theoretical foundation for further exploring the abiotic stress response of eucalyptus *EgNF-YBs*. The further understanding of the molecular regulatory mechanism of *EgNF-YBs* will accelerate the application of eucalyptus in crop yield increase.

## Data Availability

All the data and materials that are required to reproduce these findings can be shared by contacting the corresponding author.

## Supplementary Materials

The following are available online at https://www.mdpi.com/article/10.3390/plants10061107/s1, Figure S1: Phylogenetic classification of EgNF-YB gene in *Eucalyptus grandis*, Figure S2: Multiple sequence alignment of *EgNF-YB* conservative domains., Table S1: EgNF-YB gene information, Table S2: Primer sequence were used for q-PCR analysis.

## References

[1] Li W.B., Li S.C., Wang Z.K. (2012). Review of structure and function of nuclear factor Y in plant. J. Northeast. Agric. Univ..

[2] Mantovani R. (1999). The molecular biology of the CCAAT-binding factor NF-Y. Gene.

[3] Paolo B., Valentina B., Daniele M., Isaia F.L., Enrico T., Carol I. (2008). A balance between NF-Y and p53 governs the pro- and anti-apoptotic transcriptional response. Nucleic Acids Res..

[4] Zemzoumi K., Frontini M., Bellorini M., Mantovani R. (1999). NF-Y histone fold α1 helices help impart CCAAT specificity. J. Mol. Biol..

[5] Gusmaroli G., Tonelli C., Mantovani R. (2001). Regulation of the CCAAT-binding NF-Y subunits in *Arabidopsis thaliana*. Gene.

[6] Dolfini D., Gatta R., Mantovani R. (2011). NF-Y and the transcriptional activation of CCAAT promoters. Crit. Rev. Biochem. Mol. Biol..

[7] Frontini M., Imbriano C., Manni I., Mantovani R. (2004). Cell cycle regulation of NF-YC nuclear localization. Cell Cycle.

[8] Li X.Y., Mantovani R., Hooft van Huijsduijnen R., Andre I., Benoist C., Mathis D. (1992). Evolutionary variation of the CCAAT-binding transcription factor NF-Y. Nucleic Acids Res..

[9] Jennifer M. (2017). CONSTANS companion: CO binds the NF-YB/NF-YC dimer and confers sequence-specific DNA binding. Plant Cell.

[10] Lee H., Fischer R.L., Goldberg R.B., Harada J.J. (2003). Arabidopsis LEAFY COTYLEDON1 represents a functionally specialized subunit of the CCAAT binding transcription factor. Proc. Natl. Acad. Sci. USA.

[11] Riechmann J., Heard J., Martin G., Reuber L., Jiang C., Keddie J., Adam L., Pineda O., Ratcliffe O., Samaha R. (2000). *Arabidopsis* transcription factors: Genome-wide comparative analysis among eukaryotes. Science.

[12] Maere S., Bodt S.D., Raes J., Casneuf T., Montagu M.V., Kuiper M., Peer Y.V.D. (2005). Modeling gene and genome duplications in eukaryotes. Proc. Natl. Acad. Sci. USA.

[13] Petroni K., Kumimoto R.W., Gnesutta N., Calvenzani V., Fornari M., Tonelli C., Holt B.F., Mantovani R. (2012). The promiscuous life of plant NUCLEAR FACTOR Y transcription factors. Plant Cell.

[14] Laloum T., Mita S.D., Gamas P., Baudin M., Niebel A. (2013). Erratum to: CCAAT-box binding transcription factors in plants: Y so many?. Trends Plant Sci..

[15] Franco-Zorrilla J.M., Solano R. (2017). Identification of plant transcription factor target sequences. Biochim. Biophys. Acta.

[16] Ding H.X., Liu F., Zhang L.j., Yu Y.X., Wei Q. (2017). Review of structure and function of nuclear factor Y in plant. Mol. Plant Breed..

[17] Yang S., Xiao X., Yang L.H., Qian X., Chen X.J. (2014). Bioinformatics analysis of the expansin gene family in rice. Hereditas.

[18] Lotan T., Ohto M.-A., Yee K.M., West M.A.L., Lo R., Kwong R.W., Yamagishi K., Fischer R.L., Goldberg R.B., Harada J.J. (1998). *Arabidopsis* LEAFY COTYLEDON1 is sufficient to induce embryo development in vegetative cells. Cell.

[19] Liang M., Hole D., Wu J., Blake T., Wu Y. (2012). Expression and functional analysis of NUCLEAR FACTOR-Y, subunit B genes in barley. Planta.

[20] Hwang Y.H., Kim S.K., Lee K.C., Chung Y.S., Kim J.K. (2016). Functional conservation of rice OsNF-YB/YC and *Arabidopsis* AtNF-YB/YC proteins in the regulation of flowering time. Plant Cell Rep..

[21] Feng Z.J., He G.H., Zheng W.J., Lu P.P., Ming C., Gong Y.M., Ma Y.Z., Xu Z.S. (2015). Foxtail millet NF-Y families: Genome-wide survey and evolution analyses identified two functional genes important in abiotic stresses. Front. Plant Sci..

[22] Xu Z.J., Liu Y., Xu L., An D.S. (2019). bioinformatics analysis of NF-YB gene family of transcription factor in zea mays. Mol. Plant Breed..

[23] Yang M.Y., Zhao Y.J., Shi S.Y., Du X.M., Gu J.T., Xiao K. (2017). Wheat nuclear factor Y (NF-Y) B subfamily gene TaNF-YB3; I confers critical drought tolerance through modulation of the ABA-associated signaling pathway. Plant Cell Tissue Organ Cult..

[24] Su Y.S., Dong W.Y., Fu Y.H., Bo G.H., Ming G. (2015). Identification and characterization of NF-YB family genes in tung tree. Mol. Genet. Genom..

[25] Yan D.H., Fenning T., Tang S., Xia X., Yin W. (2012). Genome-wide transcriptional response of *Populus euphratica* to long-term drought stress. Plant Sci..

[26] Brooker M. (2000). A new classification of the genus Eucalyptus L’Hér. (*Myrtaceae*). Aust. Syst. Bot..

[27] Coscolin R.B.S., Broetto F., Marchese J.A., Campohermoso M.C., Paladini M.V. (2010). Effects of hydric deficiency on gas exchange parameters and metabolism of *Eucalyptus grandis* clones. Braz. J. Plant Physiol..

[28] Myburg A.A., Grattapaglia D., Tuskan G.A., Hellsten U., Hayes R.D., Grimwood J., Jenkins J., Lindquist E., Tice H., Bauer D. (2014). The genome of *Eucalyptus grandis*. Nature.

[29] Kubo T., Serizawa A. (2008). Identification, characterization and interaction of HAP family genes in rice. J. Recept. Res..

[30] Yuan G.A., Hui Z.Q., Xin C., Chu L.J. (2007). GSDS: A gene structure display server. Hereditas.

[31] Jeffares D.C., Penkett C.J., Baehler J. (2008). Rapidly regulated genes are intron poor. Trends Genet..

[32] Arnold K., Bordoli L., Kopp J., Schwede T. (2006). The SWISS-MODEL workspace: A web-based environment for protein structure homology modelling. Bioinformatics.

[33] Marco B., Stefan B., Andrew W., Konstantin A., Gabriel S., Tobias S., Florian K., Gallo C.T., Martino B., Lorenza B. (2014). SWISS-MODEL: Modelling protein tertiary and quaternary structure using evolutionary information. Nucleic Acids Res..

[34] Hackenberg D., Wu Y.F., Voigt A., Adams R., Schramm P., Grimm B. (2012). Studies on differential nuclear translocation mechanism and assembly of the three subunits of the *Arabidopsis thaliana* transcription factor NF-Y. Mol. Plant.

[35] Hang Z., Di W., Kong F., Ke L., Zhang H., Gang L. (2016). The *Arabidopsis thaliana* nuclear factor Y transcription factors. Front. Plant Sci..

[36] Shun Z.T., Dong Y., Zhong S.Z., Wu S.X., Ning T.Y., Bing S.X., Juan D.M., Yang Y.D. (2018). Bioinformatics analysis of the NF-YB gene family in rice. Mol. Plant Breed..

[37] Todeschini A.L., Georges A., Veitia R.A. (2014). Transcription factors: Specific DNA binding and specific gene regulation. Trends Genet..

[38] Maity S.N., Crombrugghe B.D. (1996). Purification, characterization, and role of CCAAT-binding factor in transcription. Methods Enzymol..

[39] Luger K., Mäder A.W., Richmond R.K., Sargent D.F., Richmond T.J. (1997). Crystal structure of the nucleosome core particle at 2.8 A resolution. Nature.

[40] Lin Y.X., Cheng Y., Jin J., Jin X.L., Cheng B.J. (2014). Genome duplication and gene loss affect the evolution of heat shock transcription factor genes in legumes. PLoS ONE.

[41] Abe H. (2003). *Arabidopsis* AtMYC2 (bHLH) and AtMYB2 (MYB) function as transcriptional activators in abscisic acid signaling. Plant Cell.

[42] Stephenson T.J., McIntyre C.L., Collet C., Xue G.P. (2007). Genome-wide identification and expression analysis of the NF-Y family of transcription factors in *Triticum aestivum*. Plant Mol. Biol..

[43] Siefers N., Dang K.K., Kumimoto R.W., Bynum W.E., Tayrose G., Holt B.F. (2009). Tissue-specific expression patterns of *Arabidopsis* NF-Y transcription factors suggest potential for extensive combinatorial complexity. Plant Physiol..

[44] Yamamoto A., Kagaya Y., Toyoshima R., Kagaya M., Hattori T. (2010). *Arabidopsis* NF-YB subunits LEC1 and LEC1-LIKE activate transcription by interacting with seed-specific ABRE-binding factors. Plant J..

[45] Nuruzzaman M., Manimekalai R., Sharoni A.M., Satoh K., Kondoh H., Ooka H., Kikuchi S. (2010). Genome-wide analysis of NAC transcription factor family in rice. Gene.

[46] Kumimoto R.W., Adam L., Hymus G.J., Repetti P.P., Reuber T.L., Marion C.M., Hempel F.D., Ratcliffe O.J. (2008). The nuclear factor Y subunits NF-YB2 and NF-YB3 play additive roles in the promotion of flowering by inductive long-day photoperiods in *Arabidopsis*. Planta.

[47] Hussey S.G., Mizrachi E., Groover A., Berger D.K., Myburg A.A. (2015). Genome-wide mapping of histone H3 lysine 4 trimethylation in *Eucalyptus grandis* developing xylem. BMC Plant Biol..

[48] Sato H., Mizoi J., Tanaka H., Maruyama K., Qin F., Osakabe Y., Morimoto K., Ohori T., Kusakabe K., Nagata M. (2014). Arabidopsis DPB3-1, a DREB2A interactor, specifically enhances heat stress-induced gene expression by forming a heat stress-specific transcriptional complex with NF-Y subunits. Plant Cell.

[49] Sato H., Suzuki T., Takahashi F., Shinozaki K., Yamaguchi-Shinozaki K. (2019). NF-YB2 and NF-YB3 Have Functionally Diverged and Differentially Induce Drought and Heat Stress-Specific Genes. Plant Physiol..

[50] Ding Y., Shi Y., Yang S. (2020). Molecular Regulation of Plant Responses to Environmental Temperatures. Mol Plant.

[51] Eddy S.R. (1998). Profile hidden Markov models. Bioinformatics.

[52] Edgar R.C. (2004). MUSCLE: A multiple sequence alignment method with reduced time and space complexity. BMC Bioinform..

[53] Zhang D., Gao F.L., Jakovli I., Zou H., Wang G.T. (2020). PhyloSuite: An integrated and scalable desktop platform for streamlined molecular sequence data management and evolutionary phylogenetics studies. Mol. Ecol. Resour..

[54] Hu B., Jin J., Guo A.Y., Zhang H., Gao G. (2014). GSDS 2.0: An upgraded gene feature visualization server. Bioinformatics.

[55] Bailey T., Elkan C. Fitting a mixture model by expectation maximization to discover motifs in biopolymers. Proceedings of the International Conference on Intelligent Systems for Molecular Biology.

[56] Bailey T.L., Mikael B., Buske F.A., Martin F., Grant C.E., Luca C., Ren J., Li W.W., Noble W.S. (2009). MEME Suite: Tools for motif discovery and searching. Nucleic Acids Res..

[57] Chen C.J., Chen H., Zhang Y., Thomas H.R., Xia R. (2020). TBtools: An integrative toolkit developed for interactive analyses of big biological data. Mol. Plant.

[58] Goodstein D.M., Shu S., Russell H., Rochak N., Hayes R.D., Joni F., Therese M., William D., Uffe H., Nicholas P. (2012). Phytozome: A comparative platform for green plant genomics. Nucleic Acids Res..

[59] Livak K.J., Schmittgen T.D. (2001). Analysis of relative gene expression data using real-time quantitative PCR and the 2^−ΔΔCT^ method. Methods.

