# Supplementary figures and images for: NF-YB-Mediated Active Responses of Plant Growth under Salt and Temperature Stress in Eucalyptus grandis
Source: Plants (Basel). 2021 May 31;10(6):1107. doi: 10.3390/plants10061107 (PMC8227622; doi:10.3390/plants10061107)

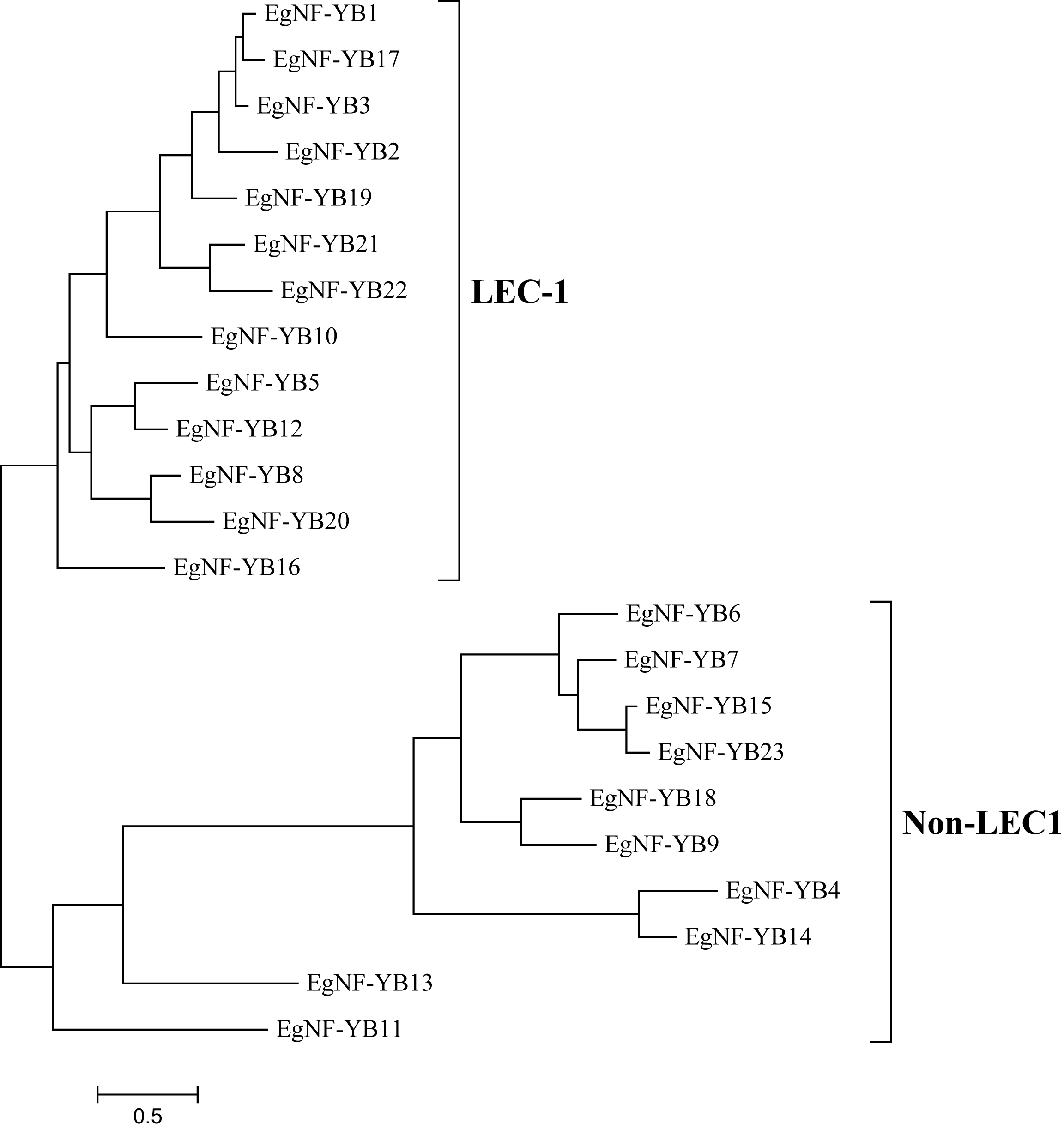

Supplement: Supplementary file 1 [file plants-10-01107-s001.zip › plants-1201885-supplementary/Supplementary Materials/Figure S1.tif]

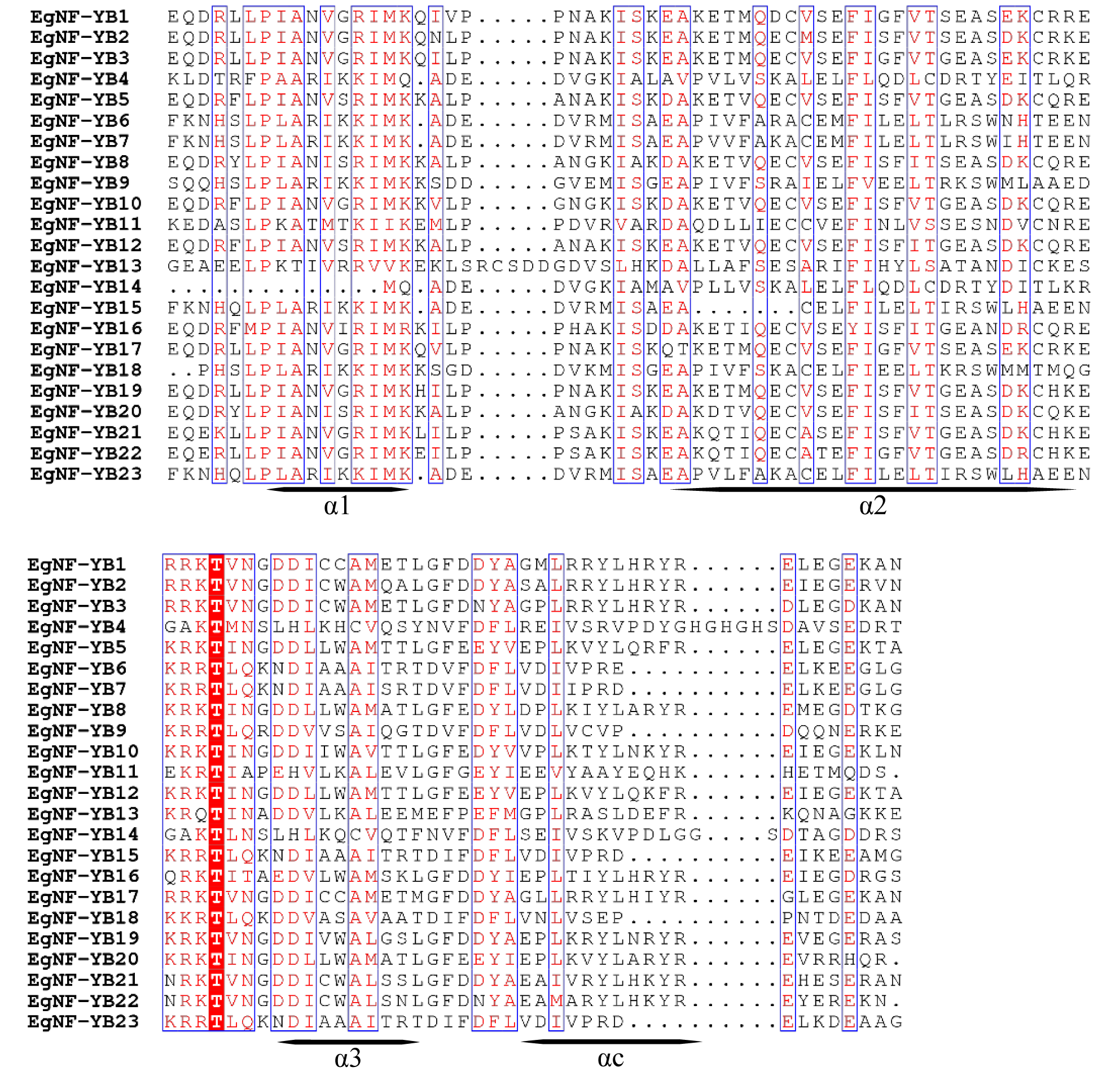

Supplement: Supplementary file 1 [file plants-10-01107-s001.zip › plants-1201885-supplementary/Supplementary Materials/Figure S2.tif]
